# TRIM64 promotes ox-LDL-induced foam cell formation, pyroptosis, and inflammation in THP-1-derived macrophages by activating a feedback loop with NF-κB via IκBα ubiquitination

**DOI:** 10.1007/s10565-022-09768-4

**Published:** 2022-10-14

**Authors:** Chao Zhu, Wei Chen, Haiming Cui, Zhigang Huang, Ru Ding, Na Li, Qinqin Wang, Feng Wu, Yanmin Zhao, Xiaoliang Cong

**Affiliations:** 1 Department of Nephrology, Changhai Hospital, Shanghai, 200433 China; 2 Department of Cardiology, Shanghai Changzheng Hospital, 415 Fengyang Road, Huangpu District, Shanghai, 200003 China; 3 Department of Cardiology, Yueyang Hospital, Shanghai University of Traditional Chinese Medicine, No. 110 Ganhe Road, Hongkou District, Shanghai, 200437 China

**Keywords:** TRIM64, Pyroptosis, NF-κB, IκBα, Atherosclerosis

## Abstract

Atherosclerosis is a chronic inflammatory disease and the main pathology behind most cardiovascular diseases and the overactivation of macrophages initiates the development of atherosclerosis. However, the specific functions of oxidized low-density lipoprotein (ox-LDL) in macrophages remain elusive. Macrophages derived from monocyte (THP-1) were treated with ox-LDL and were used to generate atherosclerosis in an in vitro model. NLRP3 inflammasome markers were examined using quantitative RT-PCR and Western blotting. Cytokines were measured using ELISA. Chromatin immunoprecipitation (ChIP) was utilized to detect nuclear factor kappa B (NF-κB) and TRIM64 interactions. A fat-rich diet was applied to ApoE-/- mice for in vivo studies. ox-LDL promoted TRIM64 expression in a time-dependent manner. According to loss- and gain-of-function analyses, TRIM64 enhanced the activation of NLRP3 inflammasomes and the expression of downstream molecules. TRIM64 directly interacted with IκBα and promoted IκBα ubiquitination at K67 to activate NF-κB signaling. We detected direct binding between NF-κB and the TRIM64 promoter, as well as enhanced TRIM64 expression. Our study revealed an interaction between TRIM64 and NF-κB in the development of atherosclerosis. TRIM64 and NF-κB formed a positive feedback to activate NF-κB pathway.

**Graphical abstract:**

ox-LDL induces foam cell formation and TRIM64 expression

TRIM64 regulates ox-LDL-induced foam cell formation, pyroptosis and inflammation via the NF-κB signaling

TRIM64 activates NF-κB signaling by ubiquitination of IκBα

NF-κB inhibition attenuates atherosclerosis in HFD-induced ApoE (-/-) mice

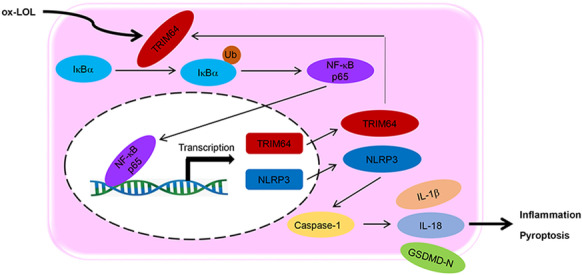

**Supplementary Information:**

The online version contains supplementary material available at 10.1007/s10565-022-09768-4.

## Introduction

Atherosclerosis is characterized by chronic thickening and loss of elasticity in the arteries. In addition, plaques accumulate in the arterial lumen, leading to atherosclerosis. A rupture of these plaques causes stroke or myocardial infarction (Zhao and Mallat 2019). Atherosclerosis is the primary contributor to cardiovascular disorders and major morbidities worldwide (Hansson and Hermansson 2011). Recently, it has been recognized that atherosclerosis is an inflammatory reaction in the vessel walls with macrophage infiltration, and the overactivation of macrophages initiates the development of atherosclerosis (Libby 2002). Foam cells derived from macrophages are the primary cells defining atherosclerosis lesions and play a critical role in plaque instability (Qian et al. 2021). Oxidized low-density lipoprotein (ox-LDL) acts via scavenger receptors, leading to pro-inflammatory signaling pathways, including NF-κB, in macrophages and vascular endothelial cells (Wang et al. 2008; Chen et al. 2018). Macrophages phagocytose excess lipids during the course of atherosclerosis, and their programmed death results in the creation of necrotic cores (Qian et al. 2021). These findings suggest that inflammation and cell death are two crucial factors in the initiation and progression of atherosclerosis.

Pyroptosis is a type of programmed cell death closely related to inflammation, which distinguishes it from apoptosis (Broz 2015). Pyroptosis is extensively involved in various diseases and inflammatory processes (Danelishvili and Bermudez 2013), and caspase-1 and gasdermin D (GSDMD) are required for canonical pyroptosis to occur (Wang et al. 2020). NLRP3 (NLR family pyrin domain containing 3) inflammasome activation is a vital mechanism regulating pyroptosis (Kaarniranta et al. 2017). NLRP3 and pro-caspase-1, alongside ASC, form inflammasomes, cleaving pro-caspase-1 to activated caspase-1 (Liu et al. 2016). Caspase-1 acts as a protease, activating GSDMD, IL-18, and IL-1β and converting them to mature forms (Broz 2015). Activated N-terminal gasdermin D (GSDMD-N) interacts with cell membranes and forms pores, disrupting cell integrity and causing pyroptosis. Pyroptosis has been found to account for a significant portion of macrophage mortality in atherosclerotic plaques as a kind of coordinated necrosis that secretes pro-inflammatory mediators (Martinet et al. 2019). Pyroptosis in the plaque generates inflammation, which results in macrophage migration and the development of foam cells. For instance, macrophages and foam cells pyroptosis mediated by NLRP3 contribute to the advancement of atherosclerosis (Qian et al. 2021). Notably, AIM2-dependent macrophage pyroptosis worsens atherosclerosis in mice (Fidler et al. 2021). However, the regulation of NLRP3 inflammasomes activation and pyroptosis in macrophage during atherosclerosis are largely unknown.

Nuclear factor kappa B (NF-κB), a vital transcription factor involved in signaling, regulates a variety of cellular processes, including inflammatory processes. NF-κB heterodimer becomes inactivated in cytoplasm and translocates to nucleus after being stimulated to attach to its target genes (Hayden and Ghosh 2004). IκBα, an NF-κB inhibitor, functions to prevent NF-κB from translocation towards nucleus (Hoesel and Schmid 2013; Schmitz et al. 2018). NF-κB activation/translocation needs the ubiquitination and degradation of IκBα. Previous studies have reported that NF-κB inhibition by pyrrolidine dithiocarbamate (PDTC) negatively regulates inflammatory responses and lipid accumulation in macrophages induced by lipopolysaccharide (Feng et al. 2014; Luo et al. 2015), indicating a viable possibility for atherosclerosis prevention and therapy. Moreover, NF-κB/NLRP3 pathway contributes to the macrophage pyroptosis in atherosclerosis (Zhang et al. 2019; Xu et al. 2021). However, their regulations in macrophage pyroptosis in atherosclerosis are not well understood.

Tripartite motif (TRIM) family proteins contribute to a wide range of biological events. TRIM family proteins a RING domain, B-box motif, and a coil-coil region. TRIM proteins are E3 ubiquitin ligase, responsible for the transfer of the ubiquitin to their targets (Li et al. 2014). The TRIM family members regulate NF-κB and IκBα signaling (Xu et al. 2019; Zhang et al. 2020). Other systems have exhibited an interaction between NF-κB/IκBα and TRIM64 (Kawai and Akira 2011). TRIM21 controls NF-κB cascades, leading to suppressed inflammatory factors in a murine model of atherosclerosis (Espinosa et al. 2009). ox-LDL promotes TRIM64 expression, suggesting a potential role of TRIM64 in atherosclerosis (Jiang et al. 2017). Therefore, we hypothesized that TRIM64 contributes to atherosclerosis through the NF-κB/IκBα pathway. In this study, TRIM64 plays a fundamental role in ox-LDL-induced cell formation, NLRP3 inflammasome, cytokine release, and NF-κB activation through IκBα ubiquitination. We established TRIM64’s function and our results shed light on future therapeutic approaches towards atherosclerosis.

## Materials and methods

### Cell culture and treatment

Human monocytic THP-1 cells (Institute of Biochemistry and Cell Biology, Shanghai Institutes for Biological Sciences, Chinese Academy of Sciences) were grown in RPMI 1640 medium, constituting 10% fetal bovine serum, 1 × 10^5^ μg/L penicillin G and 100 g/L streptomycin. Plating of cells was done at a density of 1 × 10^6^ cells/35 mm slip-covered culture dish. Incubation of THP-1 cells with phorbol myristate acetate (5 ng/mL; Sigma-Aldrich, Saint Louis, MO, USA) in the media for 24 h caused them to differentiate into macrophages. After being starved overnight, phorbol myristate acetate-differentiated THP-1 macrophages were treated with 50 μg/mL ox-LDL (Yiyuan Biotech, Guangzhou, China) in a serum-free RPMI1640 medium including 0.3% BSA for 48 h. Oil Red O staining and cellular cholesterol assays were used to determine foam cell development.

### Oil Red O staining

Cells were gently washed three–five times with PBS, fixed for 30 min in 4% formalin solution, washed with distilled water, and stained for 15 min at ambient temperature with Oil Red O (Sinopharm Chemical Reagent Co., Ltd.). Cells were then rinsed with running water. The cells were viewed under a microscope after drying. A cell was considered positive if at least five granules were spotted in it. Each treatment consisted of ten fields selected at random.

### Cellular cholesterol assay

Free cholesterol (FC) assay kit (Solarbio, Beijing, China) and total cholesterol (TC) assay kit (Elabscience Biotechnology Co. Ltd, Wuhan, China) were used to evaluate the quantities of FC and TC in cells. The following formula was used: cellular cholesteryl ester (CE) = TC-FC.

### Cell death assay

Propidium iodide (PI) and active caspase 1 staining were used to determine pyroptotic cell death. Cells (5 × 10^5^ cells/well) were cultivated in a six-well plate, reached 50% confluence, and then conditioned with Caspase-1 p20 Antibody/FITC (Eterlife Ltd., UK) and with 10 μl PI (Thermofisher) for 15 min. Accuri C6 flow cytometry (BD Biosciences, San Jose, CA, USA) was used to examine the samples.

### ELISA

Human TNF alpha (ab181421) and IL-6 (ab178013) ELISA Kits (both from Abcam), IL-18 (RAB0543) and IL-1 beta (RAB0273) ELISA Kits (both from Sigma-Aldrich) were applied to assess the release of TNF-α, IL-18, IL-1β, and IL-6 in cell supernatants following the manufacturer’s protocol. A micro-plate reader at 450 nm was applied to assess optical density.

### Dual luciferase assay

Cells were transfected with the pGL3-basic plasmid carrying the TRIM64 promoter or the pRL-TK plasmid using Lipofectamine 2000 and then incubated for 6 h at 37 °C with a vehicle or the NF-κB inhibitor (pynolidine dithiocarbamate (PDTC)). The luciferase activities were determined using dual-luciferase reporter assay kit (Promega, Madison, WI, USA) according to the supplier’s procedure.

### Chromatin immunoprecipitation (ChIP)

ChIP analysis was carried out as previously reported (Zhu et al. 2017). Briefly, cells were fixed in 1% formaldehyde, and a Bioruptor Sonicator (Diagenode; five cycles of 3 son/3 s off) was used to fragment the DNA into a sizes ranging between 200 and 1000 base pairs. The extracts were immunoprecipitated with protein A/G beads and incubated with antibodies against control IgG (Santa Cruz Biotechnology; sc-2027) or NF-κBp65 (Abcam; ab16502) at 4 °C for 12 h. The immunoprecipitates were washed, eluted, and reverse cross-linked at 65 °C for 12 h. The immunoprecipitated DNA fragment was then purified and validated using PCR (TRIM64 primers sequences: F, 5′-CCAAAGTGCTGGGATTACAA-3′ and R, 5′-CGCCACTACCCCCAACTAAT-3′).

### Cell transfection

Full length of TRIM64 was ligated into pLVX-Puro lentiviral expression vector (Clontech Laboratories, Inc., Mountain View, CA, USA). A short RNA interference sequence targeting human TRIM64 (shTRIM64-1, 5′-GGATTCAGACGACCTGCAA-3′; shTRIM64-2, 5′-GAGACAAGAAACAATCTAA-3′; shTRIM64-3, 5′-GGATAATCACTATTCAATA-3′) was cloned into pLKO.1 lentiviral vector (Addgen, USA). Co-transfection of recombinant lentiviral vectors into 293 T cells with pMD2G and psPAX2 was aided by Lipofectamine 2000. The negative control was pLVX-Puro or pLKO.1-scramble shRNA vector.

Mutant or full-length IκBα cDNA was ligated into pCMV-Tag 2B vectors, and the constructed vector was named IκBα (K22R), IκBα (K38R), IκBα (K67R), and IκBα (WT), respectively. A QuikChange site-directed mutagenesis kit (Stratagene, La Jolla, CA, USA) was used to mutation analysis. Myc-labeled TRIM64 (GENEWIZ, Suzhou, China) was ligated into p-DONR221 vector. His-tagged human ubiquitin (His-Ub) was ligated into pcDNA-DEST40. Each construct was verified by sequencing analysis. Using Lipofectamine 2000, IκBα constructs, His-Ub, and myc-TRIM64 were co-transfected into 293 T cells.

### Quantitative RT-PCR

TRIzol reagent (Invitrogen, Carlsbad, CA, USA) was used to extract total RNA. A PrimeScript III RT-PCR kit (TaKaRa, Dalian, China) was used to generate cDNA. Quantitative RT-PCR (Applied Biosystem) was conducted using an ABI 9700 real-time PCR machine (Applied Biosystems, Foster, CA, USA) using the SYBR green PCR master mix. The primers were:

- TRIM64-F: 5′-AGCCAGACAGACCAGACCTC-3′,
- TRIM64-R: 5′-TCCTCAGCAGCCCATCC-3′;
- GAPDH-F: 5′-CACCCACTCCTCCACCTTTG-3′,
- GAPDH-R: 5′-CCACCACCCTGTTGCTGTAG-3′.

Quantitative measurements were calculated using the 2^−ΔΔCT^ method.

### Western blotting

Protein was extracted using a RIPA lysis buffer including a protease inhibitor combination (Sigma-Aldrich) that had been freshly added. Supernatants were collected after centrifugation. Bicinchoninc acid reagent (Thermo Scientific) was applied to assess protein concentration. The cytoplasmic and nuclear levels of NF-κBp65 were examined using an NE-PER kit (Thermo Scientific). Sulfate–polyacrylamide gel electrophoresis (SDS-PAGE) gels (Millipore, Bedford, USA) were used to isolate the proteins and then transferred onto nitrocellulose membranes. After blocking in 5% non-fat dry milk, the membranes were then incubated overnight with the following antibodies: TRIM64 (Novus Biologicals, LLC, Centennial, CO, USA; NBP2-83,713), CD36 (Abcam; ab133625), ABCA1 (Abcam; ab18180), NF-κB (Abcam; ab16502), NLRP3 (Abcam; ab232401), ASC (Abcam; ab155970), caspase-1 (Abcam; ab207802), GSDMD-N (Abcam; ab215203), anti-IκBα (Abcam; ab32518), H3 (Cell Signaling Technology; 4499), and GAPDH (Cell Signaling Technology; 5174) followed by the horseradish peroxidase-conjugated secondary antibody. Immunoreactive band was visualized with enhanced chemiluminescence detection kit reagents (Servicebio, Wuhan, China).

### Immunoprecipitation and liquid chromatography/mass spectrometry (LC/MS) analysis

The entire TRIM64 genome was cloned into a pCMV-Flag vector. 293 T cells stably expressing Flag-TRIM64 were lysed in pre-chilled RIPA lysis buffer (1% Triton X-100, 150 mM NaCl, 20 mM Tris pH7.5). Cell lysates were treated overnight at 4 °C with anti-Flag magnetic beads (Sigma-Aldrich). Following incubation of the proteins with Flag peptide for 1 h at 4 °C, they were eluted, resolved on SDS-PAGE, and stained with Coomassie blue. As noted previously (Zhu et al. 2017), bands originating primarily from TRIM64 overexpressed cells were excised for in-gel trypsin digestion and LC/MS identification using a mass spectrometer (Thermo Scientific Q Exactive).

### Co-immunoprecipitation and ubiquitination assay

Cells were incubated in RIPA lysis buffer (containing 1 mM MgCl_2_, 100 mM NaCl, and 1 mM DTT) and with antibodies against IκBα (Abcam; ab32518), TRIM64 (Biorbyt LLC., St Louis, MO, USA; orb455353), or IgG (Santa Cruz Biotechnology, Inc.; sc-2027) in the presence of magnetic beads modified with protein A/G (New England BioLabs (Ipswich, MA, USA) for 120 min with gentle agitation at 4 °C. The beads were rinsed thrice in above indicated buffer and then evaluated by immunoblotting analysis.

### Pull-down assay

293 T cells were co-transfected with the IκBα constructs, His-Ub, and myc-TRIM64. Following 48 h of transfection, cell lysates were treated with Ni^2+^-NTA agarose beads (Qiagen). Resultant complexes were eluted by bringing them to boil in SDS sample buffer. SDS-PAGE was used to separate the proteins, and Western blotting was used to visualize the bands.

### *In vivo *experiments

Male C57BL/6 and ApoE-/- mice (Nanjing Biomedical Research Institute of Nanjing University) were fed on a normal chow diet until reaching 8 weeks of age, weighing 18–20 g. After 1 week of adaptation, ApoE-/- mice (*n* = 12) were randomly assigned to two groups: high-fat diet (HFD; purified ingredient diet, high saturated fat, 1% cholesterol, 0.25% cholic acid) + vehicle (sterile PBS) and HFD + PDTC. After ApoE − / − mice were fed with the HFD for 12 weeks, PDTC was given by intraperitoneal injection (100 mg/kg, once a day) for uninterrupted 8 weeks. The control group consisted of C57BL/6 mice (*n* = 6). The mice were weighed and sacrificed at week eight. For histological examination, abdominal aorta tissues were harvested and preserved in 4% formalin. For biochemical and immunohistochemical examination, the proteins were kept at – 80 °C. The serum levels of high density lipoprotein-cholesterol (HDL-C), low-density lipoprotein-cholesterol (LDL-C), TC, triglyceride (TG), IL-1β, IL-18, TNF-α, and IL-6 were measured using assay kits. All procedures were carried out in compliance with Shanghai Changzheng Hospital’s animal ethics guidelines and protocols.

### Statistical analysis

All measurement data were expressed as mean ± standard deviation and experiments were performed for at least three times. Statistical analysis was processed through GraphPad Prism 8.0.2 software. The one-way ANOVA was used to compute the differences between groups, followed by the Tukey post hoc test. Student’s *t*-tests were used to compare two groups. *P* < 0.05 was considered statistically significant.

## Results

### ox-LDL-induced foam cell formation and TRIM64 upregulation

**Fig. 1:**
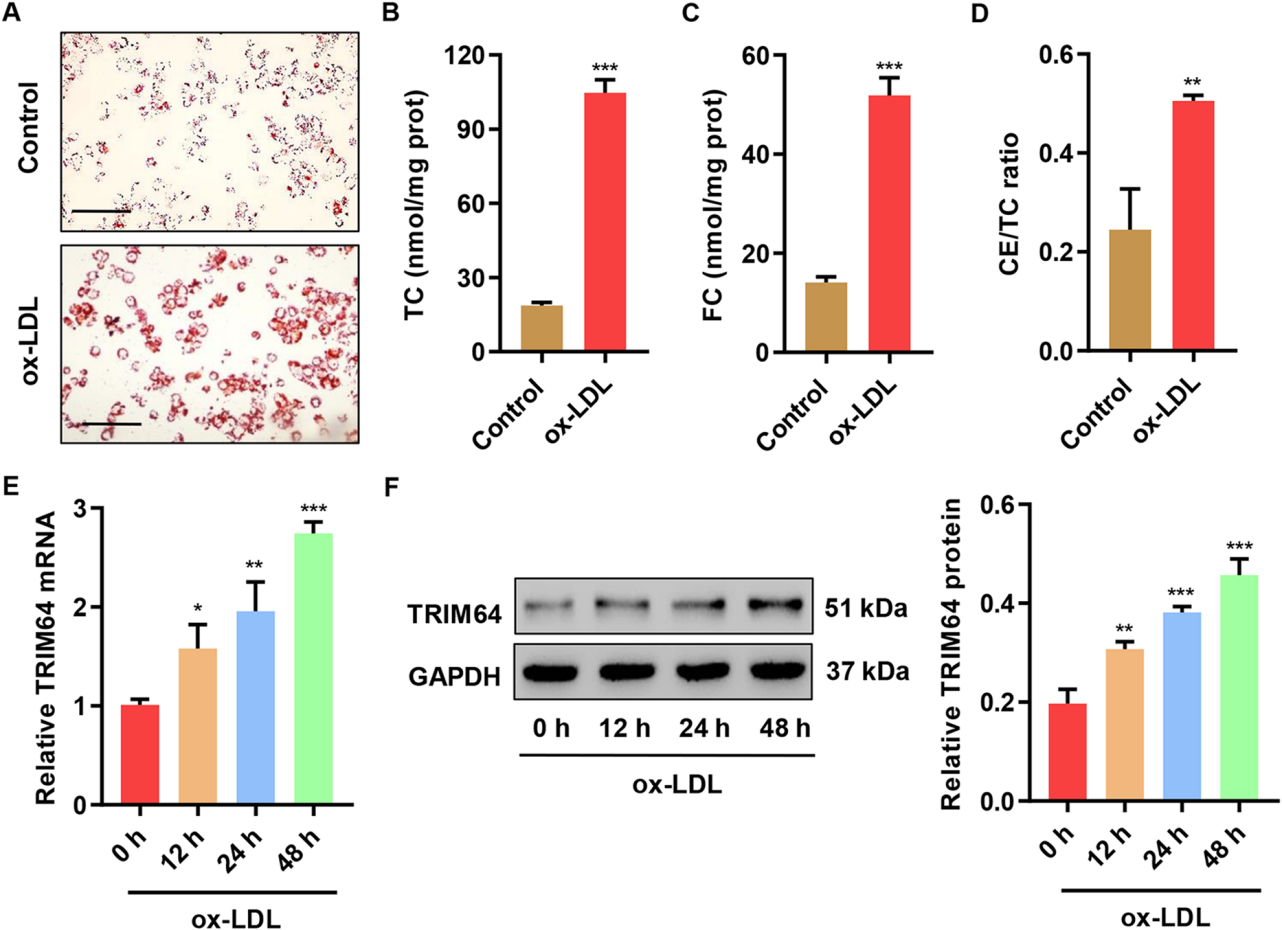
ox-LDL-induced foam cell formation and TRIM64 expression. THP-1 monocytes were primed with phorbol myristate acetate to induce macrophage differentiation. Subsequently, the macrophages were treated with or without (control) 50 mg/L ox-LDL for 48 h and then stained with **A** Oil Red O, and the **B** TC, **C** FC, **D** CE/TC levels in THP-1-derived macrophages were measured. Scale bars: 100 μm. **E**, **F** THP-1-derived macrophages were treated with 50 mg/L ox-LDL for 0, 12, 24, and 48 h, and then, TRIM64 expression was measured by quantitative RT-PCR and Western blot assays. *P < 0.05, **P < 0.01, ***P < 0.001 compared with control or 0 h. Data are expressed as mean ± SD of three biological replicates

First, using Oil Red O staining, we observed that ox-LDL-induced lipid accumulation in macrophages is derived from THP-1 (Fig. 1A). TC, FC, and CE/TC levels were significantly upregulated after ox-LDL administration in all three groups (Fig. 1B, C, and D). TRIM64 was significantly upregulated at both transcriptional and translational levels at 12 h, 24 h, and 48 h in THP-1-derived macrophages (Fig. 1E, F).

### TRIM64 suppression inhibited ox-LDL-induced foam cell formation, pyroptosis, and inflammation

**Fig. 2:**
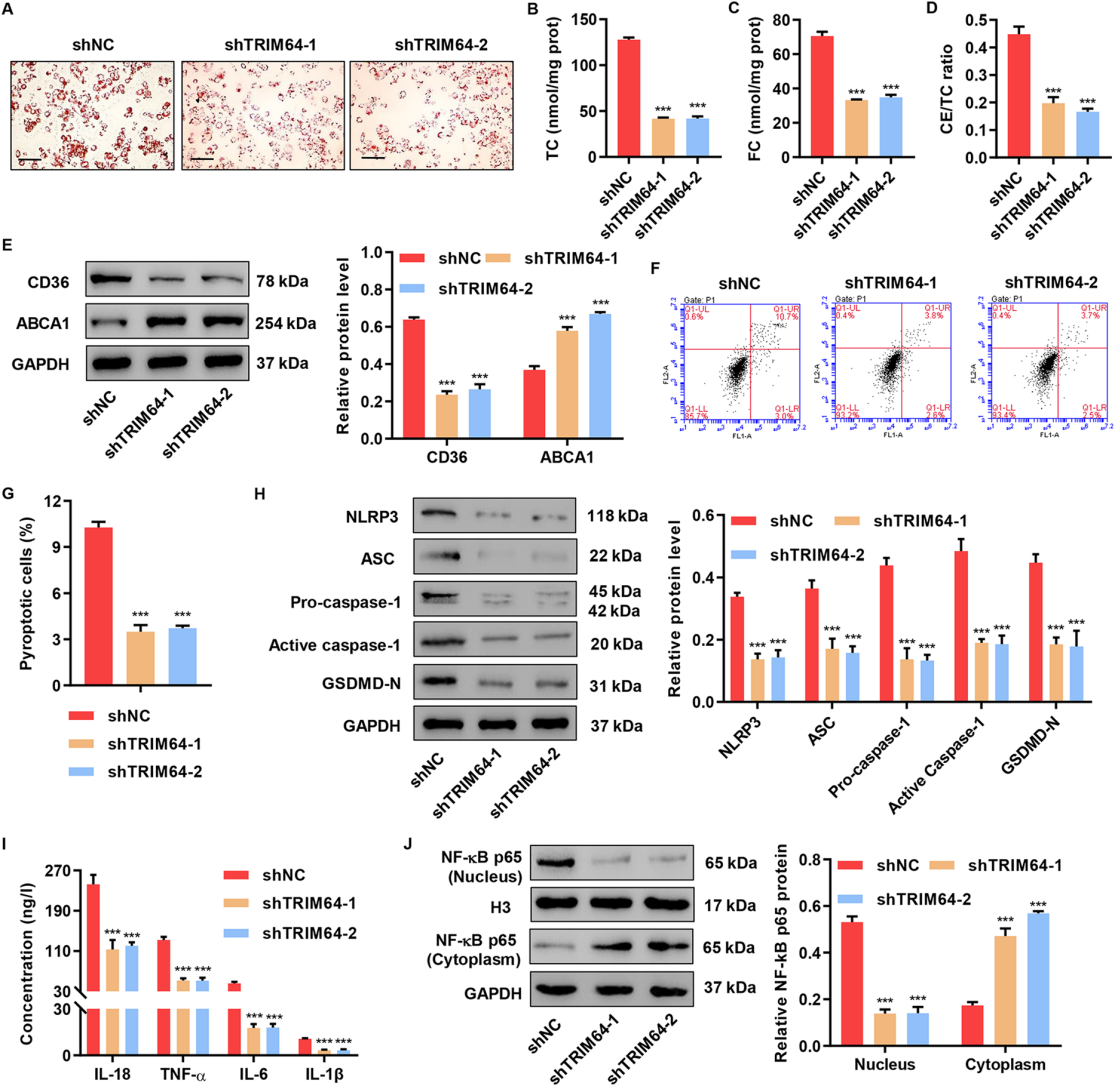
TRIM64 knockdown inhibited ox-LDL-induced foam cell formation, pyroptosis, and inflammation. THP-1-derived macrophages were transduced with TRIM64 silencing vectors (shTRIM64) or negative control (shNC) and treated with 50 mg/L ox-LDL for 48 h. **A** Cells were stained with Oil Red O. Scale bars: 100 μm. **B** TC, **C** FC, and **D** CE/TC levels were measured. **E** Western blot assays were performed to detect CD36 and ABCA1 expression. **F**, **G** Pyroptotic cell death was determined by flow cytometry. **H** Western blot assays were performed to detect NLRP3, ASC, caspase-1, and GSDMD-N expression. **I** IL-18, IL-1β, TNF-α, and IL-6 content were measured by ELISA. **J** Western blot assays were performed to detect NF-κB expression in nucleus and cytoplasm. ***P < 0.001 compared with shNC. Data are expressed as mean ± SD of three biological replicates

Previous study demonstrated that ox-LDL could promote foam cell formation and NLRP3 inflammasome activation in THP-1 macrophages (Chen et al. 2018). To examine the role of TRIM64 in atherosclerosis in THP-1 macrophages treated with ox-LDL, we first designed 3 small interference RNAs (shRNAs) targeting TRIM64. All three shRNAs suppressed the mRNA and protein expression of TRIM64 in macrophages generated from THP-1 efficiently (Figure S1A, B). shRNA-1 and shRNA-2 displayed a superior knockdown efficiency than shRNA-3; therefore, these two were chosen for subsequent assays. Next, we found suppressing TRIM64 significantly decreased ox-LDL-induced lipid accumulation in the cells (Fig. 2A). TC, FC, and CE/TC amounts were significantly decreased in the TRIM64 shRNA groups (Fig. 2B, C, and D). To determine the effects of TRIM64 on scavenger receptors and cholesterol efflux transporters, we performed Western blotting to evaluate the protein levels of CD36 and ABCA1. Our results showed that suppressing TRIM64 dramatically decreased CD36 and increased ABCA1 (Fig. 2E). PI and active caspase 1 staining were used to determine pyroptotic cell death. Suppressing TRIM64 significantly decreased cell pyroptosis (Fig. 2F, G). Next, we found that TRIM64 suppression caused a reduction in ASC, pro-caspase-1, NLRP3, GSDMD-N, and active caspase-1 compared to the control group (Fig. 2H). To explore the effect of TRIM64 on inflammatory responses in THP-1-derived macrophages treated with ox-LDL, we measured TNF-α, IL-18, IL-1β, and IL-6. We found that TRIM64 knock-down significantly reduced the expression of these factors (Fig. 2I). We next wondered whether the NF-κB pathway was affected. We found that suppression of TRIM64 abolished NF-κB activation (Fig. 2J). Our results suggest that endogenous TRIM64 is involved in foam cell formation, pyroptosis, and inflammation. We were also interested in the biological effects of TRIM64 overexpression.

### TRIM64 overexpression promoted ox-LDL-induced foam cell formation, pyroptosis, and inflammation via the NF-κB signaling pathwa*y*

**Fig. 3:**
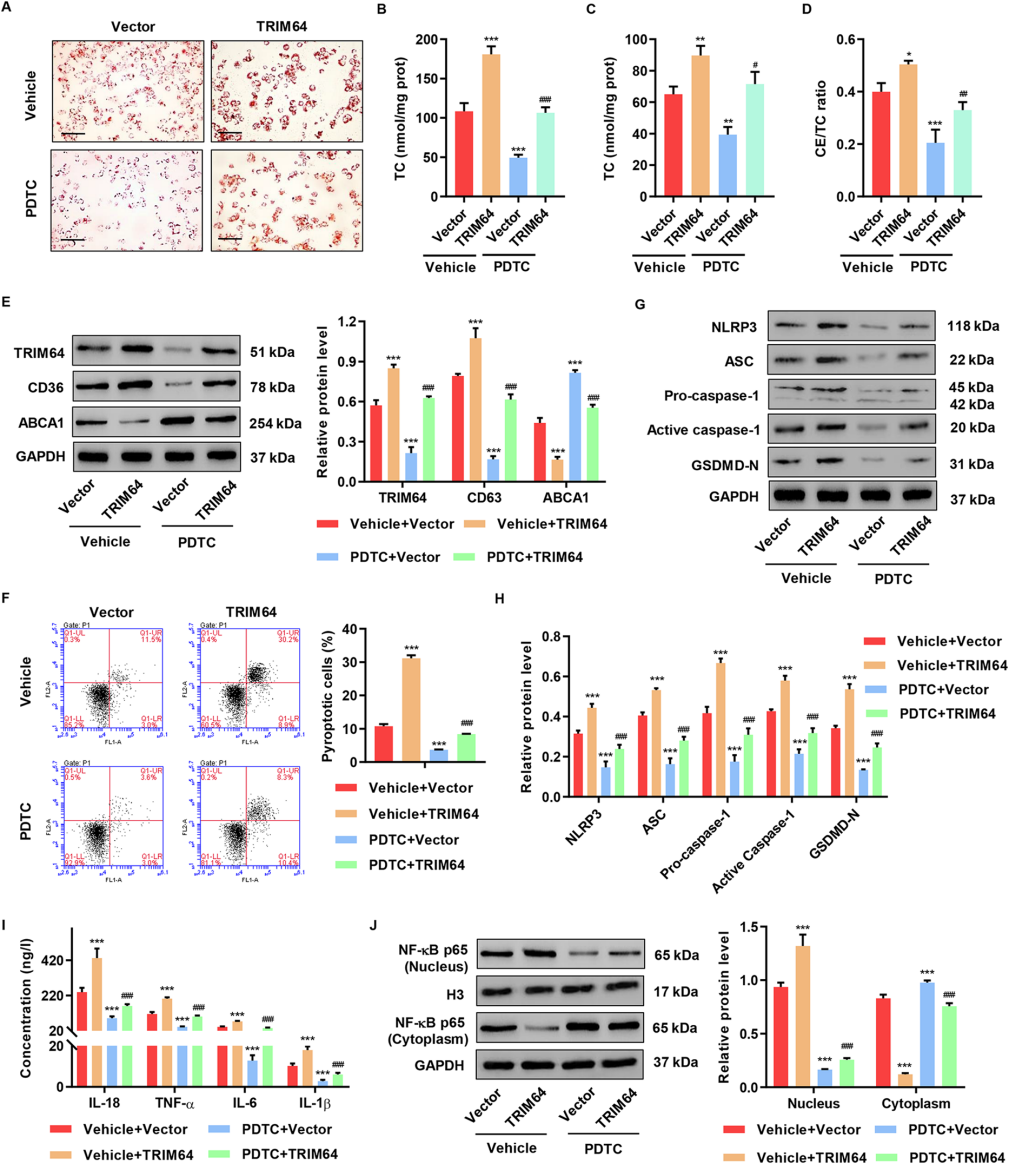
TRIM64 overexpression promoted ox-LDL-induced foam cell formation, pyroptosis, and inflammation via the NF-κB signaling. THP-1-derived macrophages were transduced with TRIM64 expressing vectors (TRIM64) or blank vector (vector) and treated with 50 mg/L ox-LDL in the presence of 10 μM PDTC or vehicle for 48 h. **A** Cells were stained with Oil Red O. Scale bars: 100 μm. **B** TC, **C** FC, and **D** CE/TC levels were measured. **E** Western blot assays were performed to detect TRIM64, CD36, and ABCA1 expression. **F** Pyroptotic cell death was determined by flow cytometry. **G**, **H** Western blot assays were performed to detect NLRP3, ASC, caspase-1, and GSDMD-N expression. **I** IL-18, IL-1β, TNF-α, and IL-6 content were measured by ELISA. **J** Western blot assays were performed to detect NF-κB expression in nucleus and cytoplasm. *P < 0.05, ***P < 0.001 compared with vector or vehicle + vector. ^#^*P* < 0.05, ^##^*P* < 0.01, ^###^*P* < 0.001 compared with vehicle + TRIM64. Data are expressed as mean ± SD of three biological replicates

We first validated the TRIM64 overexpressing plasmids, resulting in an increase in TRIM64 at transcriptional and translational levels (Figure S1C, D). Consistent with the TRIM64 knockdown results, overexpression of TRIM64 in THP-1 macrophages resulted in increased lipid accumulation (Fig. 3A). TC, FC level, and CE/TC ratio were dramatically elevated in the TRIM64 overexpression groups (Fig. 3B, C, and D). We found that overexpressing TRIM64 dramatically increased CD36 and decreased ABCA1 (Fig. 3E). TRIM64 overexpression also significantly increased cell pyroptosis (Fig. 3F). To examine the effects on pyroptosis markers, we performed Western blotting and found that TRIM64 overexpression caused an increase in ASC, pro-caspase-1, NLRP3, GSDMD-N, and active caspase-1 compared to the control group (Fig. 3G, H). We found that TRIM64 overexpression increased the levels of TNF-α, IL-18, IL-1β, and IL-6 (Fig. 3I) and also promoted NF-κB activation (Fig. 3J). NF-κB is an essential signaling pathway for atherosclerotic plaque formation. Pretreatment with a NF-κB-specific inhibitor (PDTC) notably inhibited TRIM64 overexpression-induced lipid accumulation (Fig. 3A), cholesterol content (Fig. 3B, C, and D), changes in scavenger receptor CD36 cholesterol efflux transporter ABCA1 (Fig. 3E), cell pyroptosis (Fig. 3F), and activation of ASC, pro-caspase-1, NLRP3, GSDMD-N, and active caspase-1 (Fig. 3G, H). Inflammatory response, TNF-α, IL-18, IL-1β, and IL-6 production, including NF-κB activation was significantly diminished under PDTC (Fig. 3I, J). These results suggested that TRIM64 regulates cholesterol transport, triggers pyroptosis, decreases cell viability, and increases inflammatory responses, which are all mediated by NF-κB signaling. Next, we explored how TRIM64 contributes to the NF-κB pathway.

### A positive feedback loop between TRIM64 and NF-κB p65

**Fig. 4:**
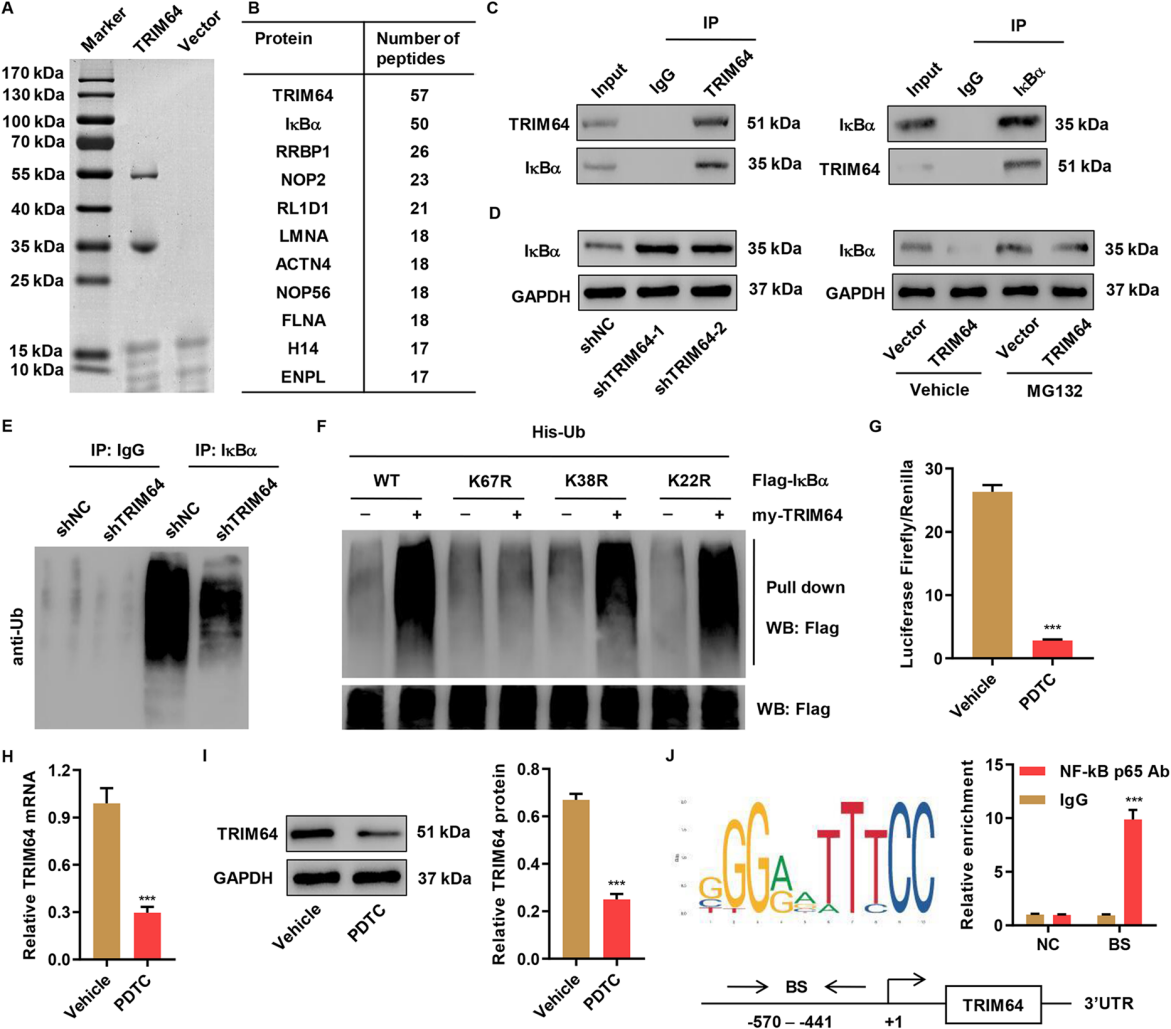
A positive feedback loop between TRIM64 and NF-κB p65. **A** Cells stably expressing FLAG-TRIM64 were generated and bound proteins were eluted with FLAG peptide, resolved by SDS-PAGE, and stained with Coomassie blue. **B** List of TRIM64-associated proteins identified by mass spectrometric analysis. **C** Cell lysates were subjected to immunoprecipitation with control IgG, anti-TRIM64, or anti-IκBα antibody. The immunoprecipitates were then blotted with the indicated antibodies. Cells were transduced with TRIM64 silencing vectors (shTRIM64) or negative control (shNC); otherwise, cells were transduced with TRIM64 expressing vectors (TRIM64) or blank vector (vector) in the absence or presence of 10 μM MG132. **D** TRIM64 expression was measured by western blot. **E** IκBα was immunoprecipitated and immunoblotted with the indicated antibodies. **F** Cells were co-transfected with the Flag-IκBα (WT) or mutant Flag-IκBα constructs (K22R, K38R, and K67R) along with myc-TRIM64 and His-Ub constructs, and the pull down assay was carried out. **G** Luciferase reporter assay was performed to evaluate the activity of the TRIM64 promoter in cells treated with PDTC or vehicle. **H**, **I** Quantitative RT-PCR and Western blot analyses of the effects of PDTC (10 μM) on TRIM64 expression. **J** The ChIP assay showed that NF-κBp65 bound to TRIM64 promoter. Top (left): NF-κBp65 binding site in the TRIM65 promoter was predicted using JASPAR database. Top (right): ChIP assays were carried out in cells. Bottom: Schematic diagram of primer for ChIP analysis. 3′UTR served as a negative control (NC). BS: binding site. ***P < 0.001 compared with vehicle or IgG. Data are expressed as mean ± SD of three biological replicates

To search for TRIM64-associated proteins, we first generated a stable cell line expressing FLAG-TRIM64. The bound proteins were eluted using anti-FLAG magnetic beads (Fig. 4A). Mass spectrophotometry analysis was performed and identified several proteins that interacted with TRIM64, including IκBα, an NF-κB inhibitor (Fig. 4B). Moreover, our results revealed that ox-LDL treatment also significantly upregulated the nuclear expression of NF-κB with a decline in cytosolic levels, and decreased IκBα expression compared with control cells, indicating their important roles during development of atherosclerosis (Figure S2A, B). Next, we performed a coimmunoprecipitation experiment and found that TRIM64 interacted with IκBα (Fig. 4C). To investigate whether TRIM64 affected IκBα expression, we downregulated and upregulated TRIM64. Our result showed that TRIM64 was negatively related to IκBα levels (Fig. 4D). Interestingly, the downregulation of IκBα by TRIM64 overexpression was blunted in the presences of MG132, the proteasome inhibitor (Fig. 4D), suggesting the involvement of the ubiquitination. Therefore, we immunoprecipitated IκBα in the absence or presence of TRIM64 shRNA. We found that knocking down TRIM64 significantly reduced IκBα ubiquitination (Fig. 4E). To identify which sites of IκBα are essential for TRIM64-mediated ubiquitination, we constructed IκBα K22R, K38R, and K67R and performed a pull-down assay. Our results illustrated that K67R completely blunted TRIM64-induced IκBα ubiquitination (Fig. 4F), suggesting K67 was the target of TRIM64. A Luciferase Reporter assay revealed that the activity of the TRIM64 promoter in cells was suppressed by PDTC (Fig. 4G). As a result, PDTC significantly reduced TRIM64 expression at transcriptional and translational levels (Fig. 4H, I). ChIP analysis revealed that NF-κB p65 bound to TRIM64 promoter (Fig. 4J).

### NF-κB inhibition attenuated atherosclerosis in HFD-induced APOE (-/-) mice

**Fig. 5:**
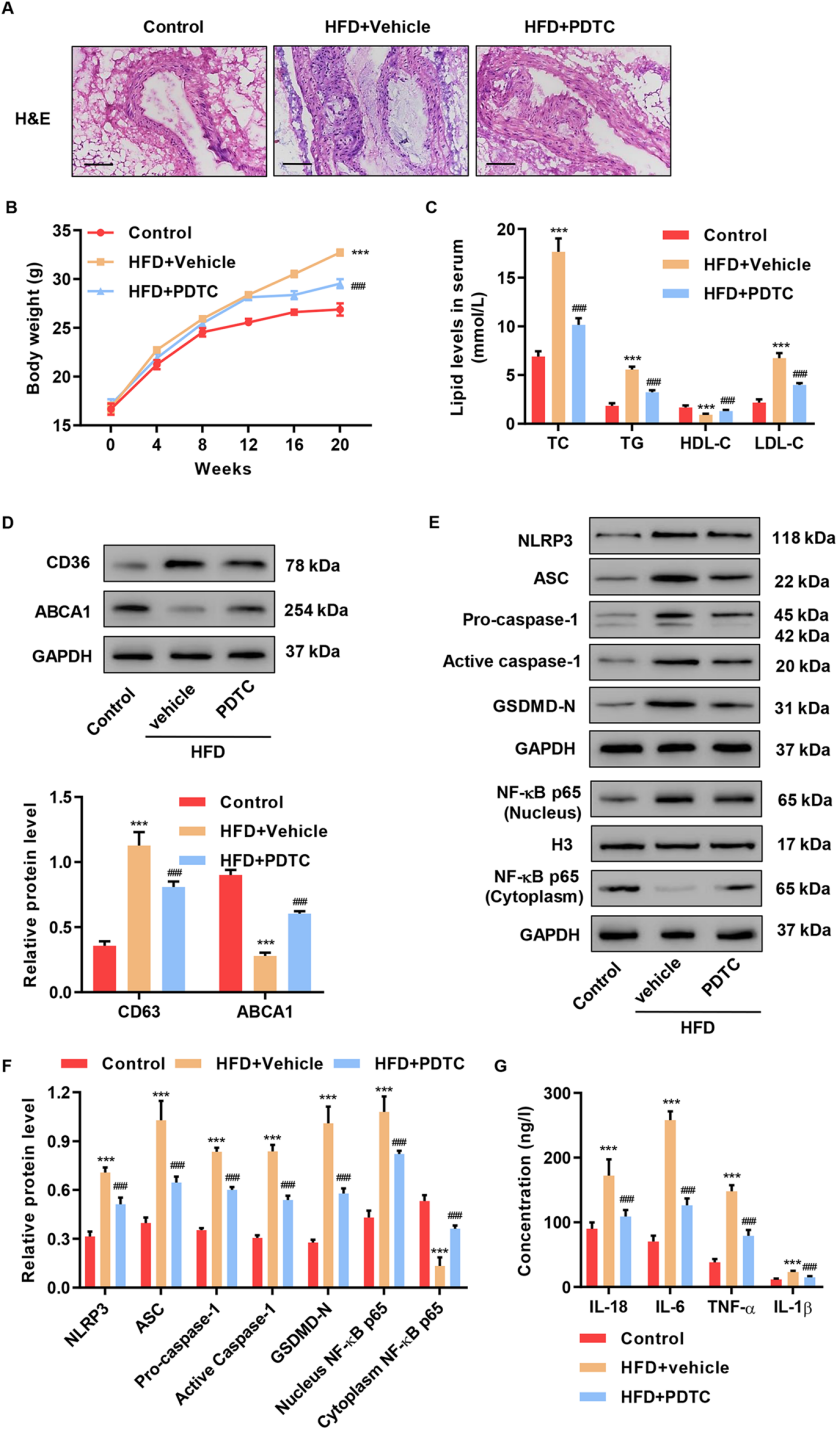
PDTC attenuated atherosclerosis in HFD-induced ApoE (-/-) mice. HFD-induced ApoE (-/-) mice were injected with PDTC or vehicle. **A** Abdominal aorta tissues were stained for H&E. Scale bars: 100 μm. **B** Body weight, **C** TC, TG, LDL-C, and HDL-C level were measured. **D**–**F** Western blot assays were performed to detect CD36, ABCA1, NLRP3, ASC, caspase-1, GSDMD-N, and NF-κB expression. **G** IL-18, IL-1β, TNF-α, and IL-6 content were measured. ***P < 0.001 compared with control. ^###^*P* < 0.001 compared with HFD + vehicle. Data are expressed as mean ± SD of six biological replicates

HFD-induced ApoE (-/-) mice were injected with PDTC or vehicle. Spontaneous atherosclerotic lesions were detected by staining aortic sinus cryosections. We found that PDTC dramatically reduced atherosclerotic lesions (Fig. 5A), body weight, TC, TG, LDL-C level, and increased HDL-C level compared to the HFD group (Fig. 5B, C). HFD increased CD36, but decreased ABCA1, whereas PDTC blunted these changes (Fig. 5D). PDTC caused a reduction in HFD-induced ASC, pro-caspase-1, NLRP3, active caspase-1, GSDMD-N, and NF-κB activation compared to the control group (Fig. 5E, F). HFD induced TNF-α, IL-18, IL-1β, and IL-6, which was inhibited by PDTC (Fig. 5G). These results imply that NF-κB inhibition significantly attenuated atherosclerosis and pyroptosis in vivo.

## Discussion

This study revealed that TRIM64, a RING type E3 ubiquitin protein ligase, was involved in foam cell formation and cytokine production through NF-κB signaling. Specifically, TRIM64 ubiquitinated IκBα at K67, ultimately resulting in increased NF-κB activity.

A recently reported gene, TRIM64 is believed to act in the innate immune responses against viral invasion. The roles of TRIM family members in cardiovascular biology are largely unknown. Atherosclerosis is considered to be an inflammatory disease because it is caused by overactivation of macrophages. Recently, an expression profile of TRIM genes was created in a study of THP1-derived macrophages activated by toll-like receptors (TLRs) (Jiang et al. 2017). TRIM64 was one of the TRIM group members that were selectively upregulated by TLR3 and TLR4 (Jiang et al. 2017). TRIM64’s roles in macrophages have not been explored. Our study is a valuable continuation in the project of TRIM profiling and we are the first group to demonstrate the molecular mechanisms of TRIM64 during macrophage activation. We have also identified that TRIM64 directly targets IκBα at K67. Consistent with our results, another TRIM member, TRIM7, is increased in atherosclerosis, and interference of TRIM7 in the same high-fat diet ApoE-/- model effectively mitigated atherosclerosis progression in a recently reported study (Ji et al. 2020). In contrast, TRIM7 controls migration and proliferation of vascular smooth muscle cells and it acts through the c-Jun/AP-1 pathway. Although suppressing TRIM64 and TRIM7 both blunted the progression of atherosclerotic lesions, the effects of these two members on lipid accumulation act on different cell types and signaling pathways. Future investigations are required to study whether TRIM64 and TRIM7 work together in atherosclerosis development.

As one of the major components of NF-κB, p65 contributes to inflammatory responses. It is well-characterized that the activity of p65 is sequestered by IκBα, and NF-κB activation in macrophages can be triggered by TLR4 as well as other stimuli. Proteasomal degradation of IκBα is required for the subsequent nuclear accumulation of p65 (Dorrington and Fraser 2019). TRIM family members are emerging essential regulators of innate immunity, notably displaying a broad range of antiviral actions (Carthagena et al. 2009). Other TRIM proteins have exhibited the ability to activate NF-κB (Uchil et al. 2013). However, TRIM64 was not one of those identified. The regulation between TRIM64 and NF-κB has not been previously studied. Since other TRIM family proteins are able to induce NF-κB signaling, we set out to examine the hypothesis that TRIM64 activates NF-κB. TRIM64 is absent in the mouse or rat genome and is specific to the human genome (Han et al. 2011). A comprehensive study on TRIM64 is lacking in all systems. Therefore, the TRIM64 localization during development of atherosclerosis and the association between NF-κB and TRIM64 in clinical samples would be further supplemented.

Pyroptosis has recently been linked to atherosclerosis (Xu et al. 2018). A study of TLR-induced NLRP3 inflammasomes in macrophages has demonstrated that NLRP3 expression is dependent on NF-κB activation at the transcription level (Qiao et al. 2012). In line with this study, we found that TRIM64 functioned as the major mediator of inflammasome formation through NF-κB and NF-κB inhibition by PDTC significantly inhibited TRIM64-induced foam cell formation, pyroptosis, and inflammation in macrophages. Consistent with our findings, PDTC treatment also inhibited the nuclear localization of NF-κΒ, pyroptosis, and proinflammatory responses in cardiomyocytes (Lei et al. 2018) and hepatocytes (Li et al. 2021). The TRIM family is one of the inflammasome receptors (Huang et al. 2019). We also confirmed that TRIM64 induced molecule expression downstream of inflammasomes and NF-κB regulated TRIM64 transcription—a feedback loop. Similar to its binding to the NLRP3 promoter, NF-κB also bound to the promoter of TRIM64. The interaction between TLRs and NF-κB is essential for the secretion of proinflammatory cytokines (Li et al. 2019). We have identified TRIM64 as the key component in the NF-κB/NLRP3 inflammasome signal pathway.

## Conclusions

In summary, our study has illuminated the relationship between TRIM64 and the NF-κB regulatory axis in atherosclerosis, suggesting that TRIM64 may serve as a potential target for future atherosclerosis therapy.

## Supplementary Information

Below is the link to the electronic supplementary material.

Supplementary file1 (DOCX 517 KB)

## Data Availability

The datasets used and/or analyzed during the current study are available from the corresponding author on reasonable request.
