# Supplementary material for: TRIM64 promotes ox-LDL-induced foam cell formation, pyroptosis, and inflammation in THP-1-derived macrophages by activating a feedback loop with NF-κB via IκBα ubiquitination
Source: Cell Biol Toxicol. 2022 Oct 14;39(3):607–20. doi: 10.1007/s10565-022-09768-4 (PMC10406714; doi:10.1007/s10565-022-09768-4)
Supplement: Supplementary file 1 — Supplementary file1 (DOCX 517 KB) [file 10565_2022_9768_MOESM1_ESM.docx]

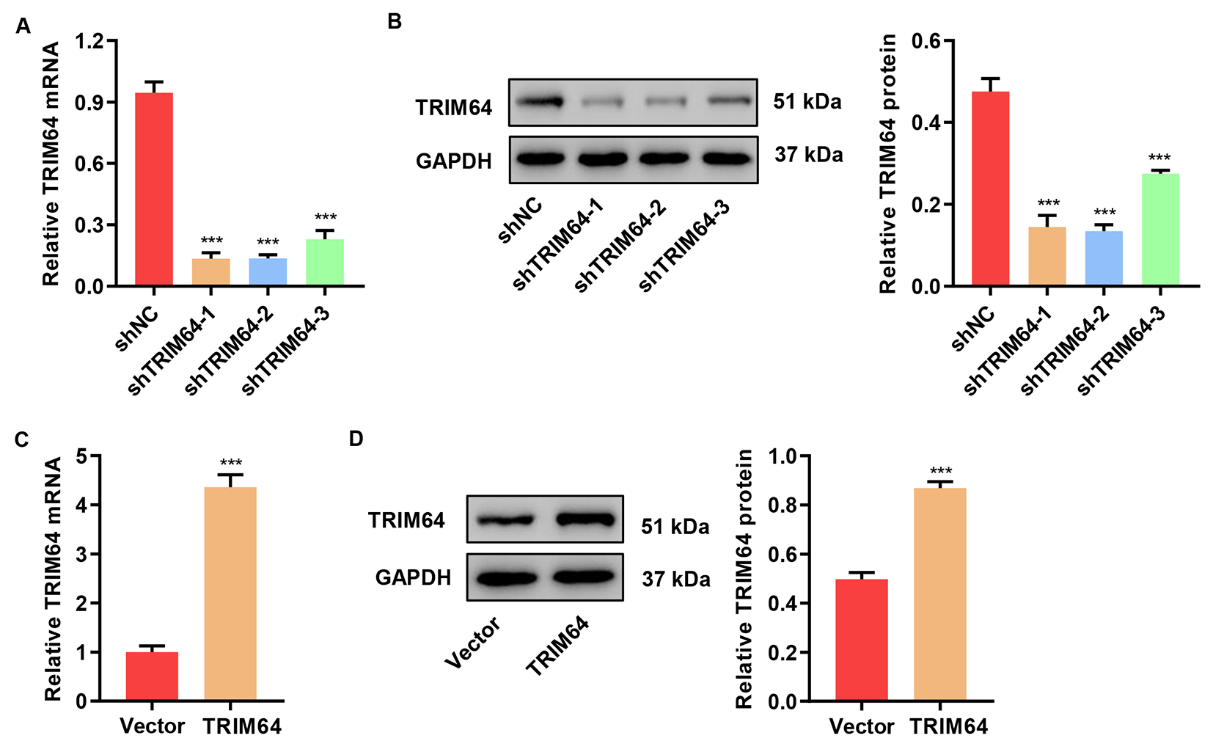


**Figure S1. TRIM65 silencing and overexpression in cells.** THP-1 monocytes were primed with 100 nM PMA for 24 h to induce macrophage differentiation. Subsequently, the macrophages were transduced with (A, B) TRIM64 silencing vectors (shTRIM64) or (C, D) TRIM64 expressing vectors (TRIM64) and the TRIM64 expression was measured by Quantitative RT-PCR and western blot. ***P<0.001 compared with shNC or vector. Data are expressed as mean ± SD of three biological replicates.


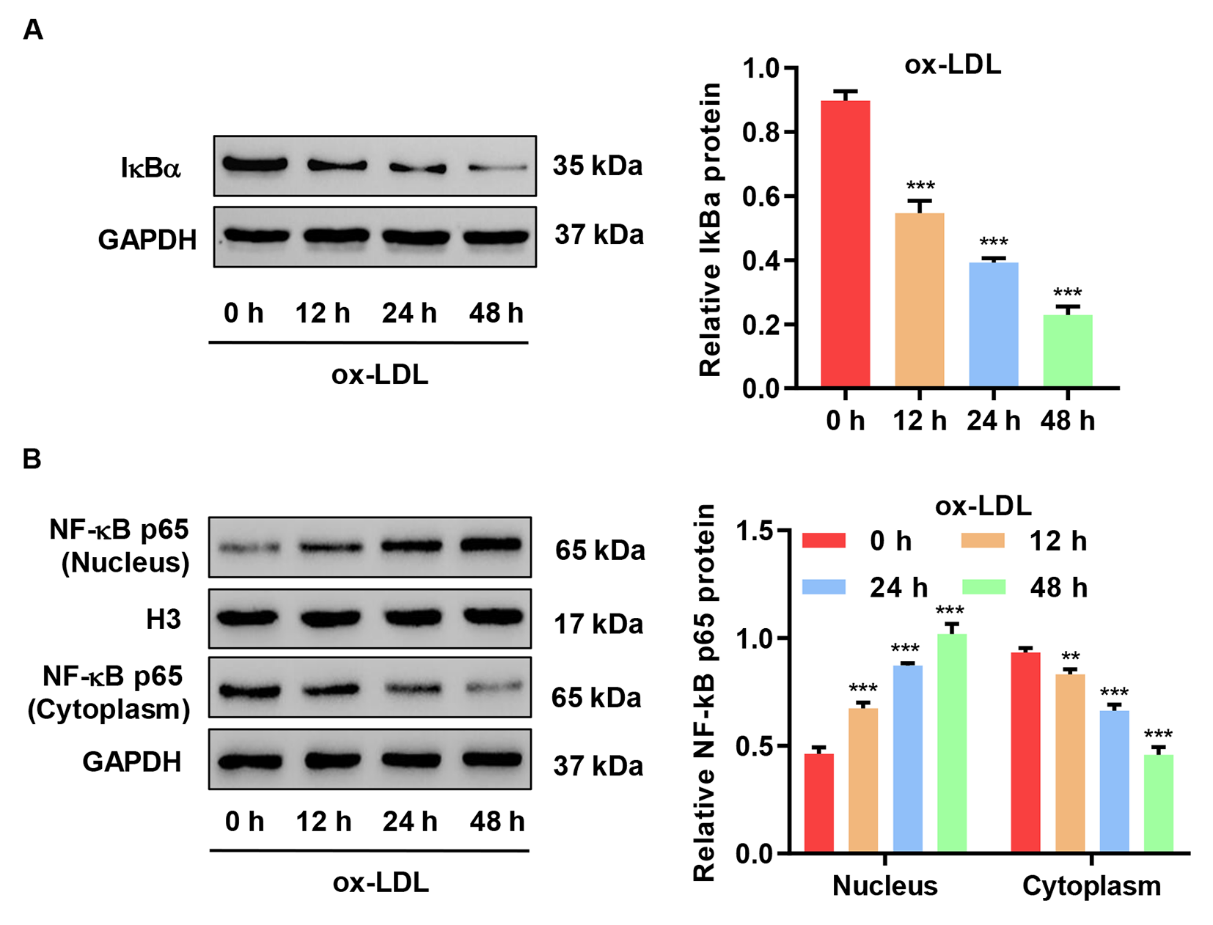


**Figure S2. IκBα and NF-κB expression in cells.** THP-1-derived macrophages were treated with 50 mg/l ox-LDL for 0, 12, 24, and 48 h and then (A) IκBα and (B) NF-κB expression was measured by Western blot assays. **P<0.01, ***P<0.001 compared with 0 h. Data are expressed as mean ± SD of three biological replicates.
